# Phase-plastic aggresomes as tunable regulators of bacterial dormancy depth

**DOI:** 10.1128/msystems.00492-25

**Published:** 2026-06-12

**Authors:** Jun Yang, Yujia Xian, Chenyi Wang, Yingying Pu

**Affiliations:** 1 State Key Laboratory of Oral & Maxillofacial Reconstruction and Regeneration, Key Laboratory of Oral Biomedicine Ministry of Education, Hubei Key Laboratory of Stomatology, School & Hospital of Stomatology, Medical Research Institute, Wuhan University 619779 , Wuhan, China; 2 Frontier Science Centre for Immunology and Metabolism, Wuhan University, Wuhan, China; Broad Institute, Cambridge, Massachusetts, USA

**Keywords:** cell dormancy, persister, aggresome, phase separation, dormancy depth

## Abstract

Bacterial persistence, a major clinical challenge in chronic and biofilm-associated infections, is driven by a dormant subpopulation capable of surviving antibiotic treatment without acquiring genetic resistance. This review summarizes recent advances supporting a unifying model in which phase-plastic aggresomes—protein-RNA condensates formed via liquid-liquid phase separation—function as tunable regulators of bacterial dormancy depth. We propose that the material state of aggresomes, ranging from liquid-like to gel-like, governs cellular recovery kinetics by modulating the sequestration and availability of core cellular machinery. Under stress, aggresomes dynamically assemble in response to triggers such as ATP depletion and macrophage-derived reactive oxygen species, enabling metabolic arrest while preserving viability. Their composition evolves over time, initially favoring reversible components that support shallow dormancy, then maturing into more gel-like states that deepen dormancy and delay resuscitation. This physical continuum allows bacteria to adaptively manage fitness trade-offs between survival and recovery. We further explore how aggresome plasticity opens new therapeutic avenues for bacterial persistence, including strategies to dissolve or solidify these condensates. Understanding aggresomes as adaptive organelles offers a transformative perspective on bacterial persistence and identifies novel targets for combating recalcitrant infections.

## INTRODUCTION

Persisters represent a heterogeneous subpopulation of dormant cells that can evade antibiotic killing without acquiring genetic resistance, making them particularly difficult to eradicate. This survival mechanism is a major cause of recurrent and chronic infections, including tuberculosis and biofilm-associated diseases (1–4). This dormant state is not merely a mechanism for antibiotic tolerance; rather, it functions as a broader survival strategy against various environmental stressors. These stressors include nutrient depletion, oxidative stress, and host immune defenses, all of which can trigger bacteria to enter a protective, non-replicating state (5–7). While the phenomenon of bacterial dormancy is well-recognized and extensively studied, a critical knowledge gap exists in quantitatively linking the observed heterogeneity in persister cell behavior to specific molecular effectors that govern their distinct physiological states. Addressing this gap is crucial for developing effective strategies to combat persistent infections. A deeper understanding of the molecular mechanisms underlying the varying depths of dormancy could enable the development of novel therapeutic approaches aimed at either awakening these dormant cells to render them susceptible to antibiotics or driving them into an irreversible non-viable state.

This review proposes that the formation and material properties of phase-plastic aggresomes, which are protein-RNA condensates, offer a physical basis for a tunable “dormancy depth,” providing a unifying framework to understand the intricate mechanisms underlying bacterial survival and subsequent recovery from persistent states (8–11). By exploring the biophysical characteristics of aggresomes, we aim to shed light on how their tunability influences the varying degrees of dormancy observed in persistent bacterial populations.

## BACTERIAL DORMANCY: A SURVIVAL STRATEGY UNDERMINING ANTIMICROBIAL THERAPY

Bacterial dormancy constitutes a fundamental survival strategy, which enables bacteria to withstand severe environmental stresses by substantially reducing metabolic activity while preserving cellular viability, thereby facilitating resuscitation when environmental conditions become favorable again (12–14). Notably, dormancy promotes population resilience under stress through the generation of phenotypic heterogeneity (15), which presents a major challenge to the effective management of clinical infections. Specific dormant subpopulations—particularly persister cells and viable-but-nonculturable (VBNC) cells—play a pivotal role in the development of chronic and recurrent infections and contribute significantly to therapeutic failure due to their inherent tolerance to conventional antibiotic treatments (16–19). These cells serve as persistent reservoirs that evade elimination by standard therapies, which are largely ineffective against cells exhibiting reduced metabolic activity and limited antibiotic target engagement (19–21). Upon cessation of antibiotic exposure or in response to favorable changes in the host environment, resuscitated dormant cells can reinitiate infection cycles, resulting in clinical relapses and further contributing to disease persistence (22, 23). This persistence-resuscitation cycle highlights dormancy as a key mechanism that undermines therapeutic effectiveness and sustains bacterial pathogenicity within the host (24, 25).

Clinically, this phenomenon is evident in cystic fibrosis lung infections, where dormant bacterial pathogens remain undetectable by standard diagnostic methods and resist antibiotic treatment, thereby facilitating persistent colonization and recurrent disease exacerbations (26). Similarly, biofilm-associated infections on medical devices are maintained by dormant bacterial subpopulations capable of withstanding high concentrations of antibiotics, thus contributing to the chronic nature of infections in implant-related contexts such as catheters and prosthetic joints (27). Moreover, chronic wound infections are characterized by the presence of dormant bacteria that contribute to treatment resistance and disease relapse, often through mechanisms involving profound dormancy accompanied by alterations in cellular integrity (28, 29).

Bacterial dormancy can be induced by a diverse array of environmental stressors through specific molecular mechanisms. Nutrient starvation—particularly in terms of carbon, nitrogen, or amino acid scarcity—induces dormancy primarily through the stringent response pathway mediated by the second messenger (p)ppGpp. This pathway reprograms metabolism toward maintenance and is activated by sensors such as RelA (21, 30, 31). Sub-lethal exposure to antibiotics, especially those targeting cell walls like β-lactams, triggers protective dormancy pathways that often involve the activation of toxin-antitoxin (TA) modules and a reduction in cellular activity to evade lethal effects (18, 19, 22, 29). Host defenses—including reactive species induced by phagocytosis (reactive oxygen species [ROS]/RNS), phagolysosomal acidity, niche nutrient restriction, and antimicrobial peptides—serve as potent inducers of dormancy. These factors compel pathogens such as *Mycobacterium tuberculosis* and *Salmonella* into latent states to withstand immune attacks (32–35). Furthermore, environmental extremes—including osmotic shifts, heat/cold shock, hypoxia/anoxia, and desiccation—promote dormancy through species-specific pathways involving sigma factors or TA systems (32, 36, 37). Importantly, dormancy frequently represents a preemptive adaptation to sub-lethal stress signals that enables tolerance without necessitating genetic mutations. Elucidating the molecular sensors that connect these diverse triggers to the establishment of dormancy is essential for developing therapeutic strategies aimed at blocking entry into this state or forcing resuscitation while sensitizing dormant cells to conventional antibiotics.

## DORMANCY DEPTH: A CONTINUUM OF REVERSIBILITY

Historically, dormancy was often viewed in binary terms: a cell was either active or dormant. However, emerging research establishes that dormancy is a spectrum of metabolic activity and physiological states (5, 8, 38). This mirrors findings in yeast spores, where dormancy depth has been shown to behave as a continuous and quantifiable spectrum governed by physical constraints on intracellular dynamics (39). Cells can occupy various positions along this continuum, from a shallow dormancy with moderately reduced metabolism to an extremely deep state of quiescence. The position on this continuum dictates critical phenotypic outcomes, most notably the time required for the cell to resuscitate and resume growth once favorable conditions return. This continuum model reconciles the observed diversity of dormant states, from quickly reviving persisters to the deeply dormant, VBNC cells. “Shallow” persisters may quickly resume growth after the removal of stress and can be classified as persister-fast-recovery (Persister-FR). In contrast, “deep” persisters demonstrate prolonged lag phases and exhibit tolerance to multiple classes of antibiotics, categorizing them as persister-slow-recovery (Persister-SR). VBNC cells represent an even deeper state of dormancy, necessitating specific resuscitation signals while remaining unable to grow on conventional culture media despite maintaining detectable metabolic activity. This phenotypic variation enables bacterial populations to survive diverse threats, with dormancy depth functioning as an adaptive mechanism that allows evasion of environmental pressures such as antibiotics (4, 5, 8).

Based on these observations, we utilized the term “dormancy depth” to quantitatively measure the extent of dormancy, with the primary criterion being the lag time for resuscitation after stress removal. It represents the magnitude of the physicochemical and physiological barriers that a dormant cell must overcome to return to an active, growing state. A cell with shallow dormancy depth faces minimal barriers and can resuscitate quickly, while a cell with great dormancy depth is trapped behind significant hurdles, leading to a prolonged lag time or even an irreversible exit from the cell cycle. The resuscitation process can be conceptualized by the framework lag time = dormancy depth/escaping speed, where “escaping speed” is the cell’s innate capacity to dismantle these barriers, dependent on factors like residual ATP levels and enzymatic activity. In this context, Persister-FR cells exhibit a shallow dormancy depth; their metabolic activity, while reduced, is sufficient to power a rapid “escaping speed,” resulting in short lag times (<12 h). In contrast, Persister-SR cells possess a high dormancy depth, often resulting from more severe or prolonged stress (>12 h). They require more time and energy to resuscitate and may be the subpopulation responsible for extended treatment cycles and late relapses in infections (8). The primary driver pushing a persister deeper along this continuum is the depletion of intracellular ATP, which acts as a master regulator of cellular energy and the solubility of biomolecules. In practice, dormancy depth can also be experimentally approximated through additional measurable parameters, including the probability of resuscitation upon stress removal, intracellular ATP replenishment kinetics, and the timing of transcriptional and translational reactivation. Importantly, dormancy depth is not only determined by the nature of the stress but also by its duration, as prolonged stress can drive the maturation of aggresomes from liquid-like to more gel-like states.

## THE ASSOCIATION BETWEEN DORMANCY DEPTH AND AGGRESOME

**Fig 1:**
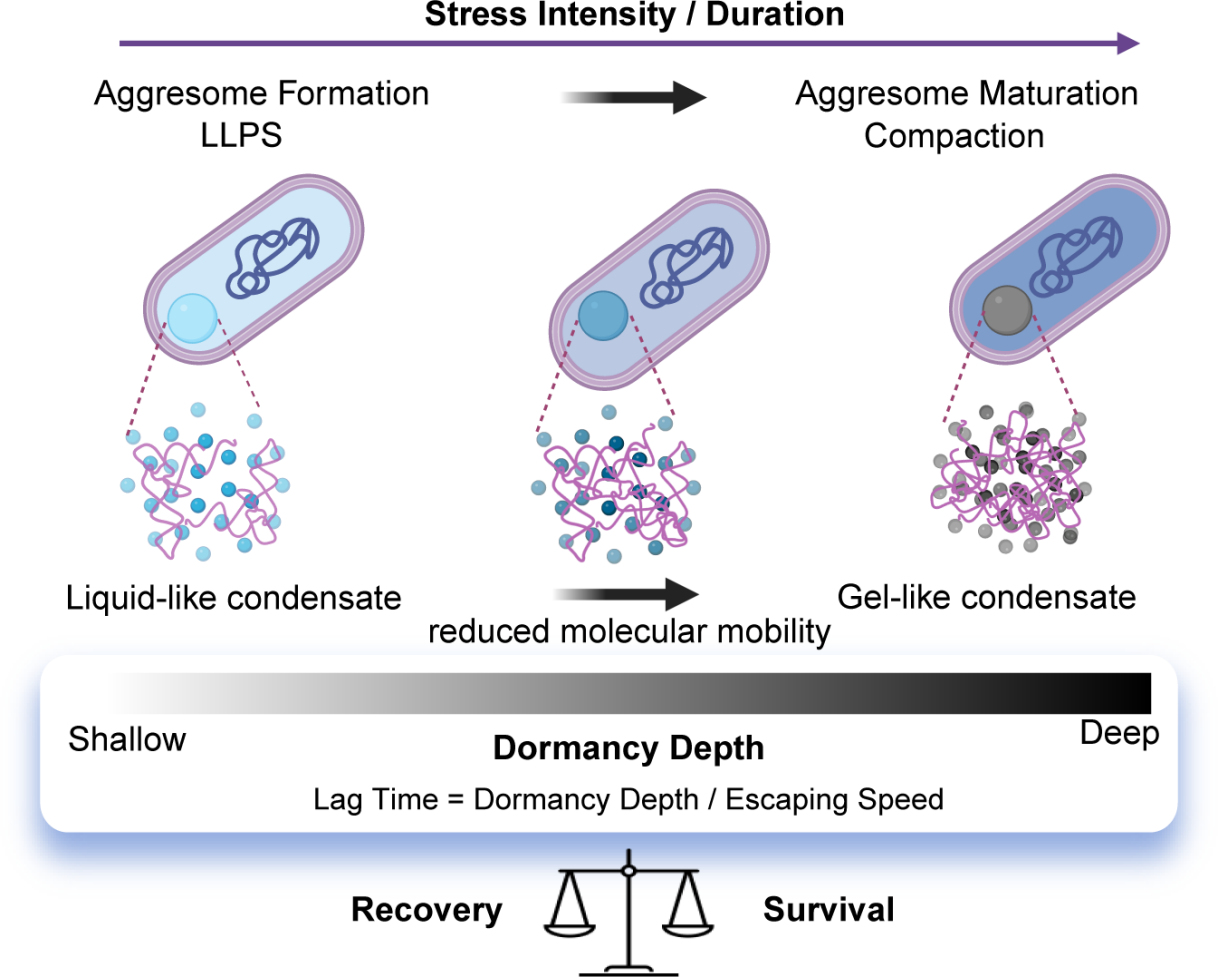
Phase-plastic aggresomes as tunable regulators of bacterial dormancy depth. Bacteria form protein-RNA condensates (aggresomes) through liquid-liquid phase separation. Their physical states, ranging from liquid-like to gel-like, regulate bacterial dormancy depth: liquid-like states favor resuscitation, while gel-like states deepen dormancy and delay recovery. This reversible system allows bacteria to balance survival and resurgence, offering new targets for combating drug-resistant bacteria.

The physical embodiment of dormancy depth is found in the formation and material state of aggresomes (8–11). Aggresomes are dynamic, protein-based condensates that form in response to proteostatic stress, often visible as dark foci within the cell. They are not static aggregates but undergo a stress-dependent material state transition that directly correlates with dormancy depth. Our study revealed that in shallow dormancy with short lag times, aggresomes remain in a liquid-like state with high fluidity (fluorescence recovery after photobleaching [FRAP] recovery time ~50 s). This presents a low physicochemical barrier to disaggregation, resulting in a short lag time during resuscitation. Under prolonged stress, these structures compact into a gel-like or solid-like state with low fluidity (FRAP recovery time >300 s). Experimental evidence has indicated that the fluidity of the aggresomes is positively correlated with the recovery rate (*R*² = 0.87). This gel-like aggresome represents a high dormancy depth, creating a significant kinetic trap for essential cellular machinery. This state characterizes deeply dormant cells with prolonged lag times or, in extreme cases, very low probabilities of resuscitation, making resuscitation a rare stochastic event. Therefore, the disassembly of aggresomes upon stress removal is a critical step governing the “escaping speed” and is a central process in determining whether a dormant cell can successfully traverse back along the continuum to an active life (Fig. 1).

## AGGRESOMES: MEMBRANELESS ORGANELLES FORMED BY LIQUID-LIQUID PHASE SEPARATION (LLPS)

Initial differential interference contrast microscopy of persister cells revealed the consistent presence of dark foci, which we termed aggresomes, predominantly localized at one or both poles of the rod-shaped cells (8). Subsequent characterization demonstrated that these bacterial aggresomes are not merely amorphous protein aggregates; rather, they are dynamic, functional, membraneless organelles formed through LLPS (9, 10). Their formation is initiated by various environmental stressors, including ATP depletion, which diminishes ATP hydrotropic activity and leads to a breakdown of proteostasis. Additionally, ROS, often encountered during macrophage phagocytosis, are significant triggers for aggresome biogenesis (11). The underlying physical principle for this transition involves a net increase in favorable attractive protein interactions, where interaction energies (𝜖_AA_) surpass approximately 1.8 𝑘_𝐵_𝑇 under thermal equilibrium. These condensates exhibit characteristic liquid-like properties, such as rapid FRAP within roughly 50 s, the ability to fuse with other droplets, and sensitivity to 1,6-hexanediol, an aliphatic alcohol known to disrupt weak hydrophobic interactions. *In vivo*, aggresomes typically present as spherical structures with a diameter of about 300 nm and frequently localize to the cell poles (9, 10).

Beyond their physicochemical characteristics, aggresomes play a crucial role in bacterial fitness and the development of antibiotic persistence. During periods of stress, these organelles sequester a diverse array of proteins and RNAs, including those involved in metabolism and ribosomal function, thereby inducing a dormant state characterized by reduced ATP levels and suppressed metabolic activity. This dormancy allows bacteria to tolerate antibiotic treatments while retaining the capacity to resuscitate once the environmental stress subsides. In *Salmonella*, for instance, reactive oxygen species produced by macrophages promote aggresome formation, which significantly increases the frequency of antibiotic-persister cells. These dormant bacteria upregulate genes associated with the *Salmonella* pathogenicity island 1 (SPI-1) type III secretion system (T3SS) and then resume growth via SPI-2 T3SS expression as ROS levels decline, facilitating recurrent infection (11).

Bacterial aggresomes are distinguishable from other LLPS-driven condensates, including transcriptional hubs, RNA degradosomes, and stress granule-like assemblies. While all such biomolecular condensates assemble through LLPS and contribute to cellular organization and stress adaptation, aggresomes are specifically induced under proteotoxic or energy-depleting conditions (e.g., ATP depletion, heat shock, or antibiotic exposure). They are characterized by the selective sequestration of misfolded proteins, molecular chaperones, and long RNAs, while tending to exclude certain negatively charged ribonucleases through electrostatic interactions (10, 40). In contrast, RNA degradosomes (such as BR-bodies formed by RNase E in *Caulobacter crescentu*s) are RNA-dependent structures that facilitate compartmentalized RNA degradation and disassemble upon RNA cleavage (41). Transcriptional hubs enhance gene expression under both basal and stress conditions but lack the protein-aggregation properties characteristic of aggresomes (42, 43). Although bacterial stress granule-like structures may share functional overlap with aggresomes, they are not defined by identical compositional or dynamic traits (44).

According to our proposed framework, an aggresome is empirically defined by three core criteria: (i) dynamics—formation at defined intracellular locations, spatial localization within the bacterial cytoplasm (often near the cell poles), reversible assembly/disassembly, and a functional role in promoting post-stress recovery; (ii) composition—enrichment of misfolded proteins, chaperones, and specific long RNAs, with exclusion of negatively charged ribonucleases; and (iii) triggers—induction by proteotoxic or energetic stress, such as ATP depletion or heat shock.

Ultimately, bacterial aggresomes represent a conserved LLPS-driven adaptive strategy that links biomolecular condensation to the regulation of dormancy depth and persistence. This mechanism illustrates how even prokaryotic organisms leverage biomolecular condensation to navigate and thrive in hostile environments. The ability to form such dynamic, reversible organelles provides bacteria with a critical survival advantage, allowing them to endure transient adverse conditions and subsequently resume growth when favorable conditions return. While other bacterial condensates contribute to cellular organization and stress responses, the dormancy-depth framework proposed here is primarily based on aggresome biology, which currently provides the most direct experimental link between condensate material state and bacterial dormancy.

## COMPOSITIONAL PLASTICITY DICTATES AGGRESOME FUNCTION

The functional role of aggresomes is not static but is dynamically dictated by their evolving composition, which follows a temporal sequestration hierarchy that mirrors the cell’s strategic shutdown and preservation processes (8, 10). This compositional plasticity is key to their function as tunable regulators of dormancy. During the initial phase of stress, the aggresome primarily incorporates proteins central to high-energy-consuming processes, such as ribosomal subunits for translation and metabolic enzymes for ATP production. The early sequestration of the protease HslU is particularly notable, as it may represent a mechanism to temporarily halt protein turnover and conserve resources (8–10).

Critically, this protein-centric view has been expanded by the discovery that aggresomes also function as protected reservoirs for mRNA (10). As demonstrated by Pei et al., specific transcripts, particularly longer mRNAs encoding functions such as ribosome and fatty acid biosynthesis, are selectively enriched within aggresomes under stress (Table 1). Importantly, RNA may serve dual roles within aggresomes: a protective role by preserving transcripts during stress, and an architectural role by contributing to the structural stability and organization of the condensate. This recruitment is not merely correlative but is driven by fundamental biophysical principles, as modeled by Flory-Huggins theory, where longer RNA chains gain sufficient favorable enthalpic interactions with aggresome proteins to overcome entropic penalties. This establishes the aggresome as a ribonucleoprotein condensate from its earliest stages.

**TABLE 1:** Aggresome components as determinants of phase plasticity and dormancy depth^*a*^

Protein/component	Function	Aggregation kinetics and context	Phase state influence and functional role
HsIU	ATP-dependent protease subunit	Fast (≤60 min); early responder to ATP depletion.	Liquid-state promoter. Its early entry helps nucleate condensates while maintaining initial reversibility (1).
Ribosomal proteins and rRNA	Protein translation	Early (e.g., 16 h starvation); major component of the protein-rich core.	Gelation promoter. High abundance and multivalency drive a liquid-to-solid transition, deepening dormancy (2).
DnaK	Hsp70 chaperone, disaggregase	Variable; recruited to pre-formed aggresomes, especially during recovery.	Disaggregation trigger and plasticity regulator. ATP-dependent activity reverses aggregation, fluidizing the condensate to enable resuscitation (2).
mRNA (long transcripts)	Genetic information	Enriched under stress; selectively partitioned via length-dependent affinity.	Liquid-state modulator and stability factor. Contributes to condensate volume and protects transcripts from cytosolic ribonucleases (3).
RNA polymerase	Transcription	Incorporated during prolonged stress.	Metabolic shutdown effector. Sequestration halts global transcription, reinforcing the dormant state (3).
RNases (e.g., RNB, RBN)	mRNA degradation	Actively excluded due to negative surface charge.	Protective barrier. Electrostatic exclusion creates a safe haven for mRNA, preserving the translatome for recovery (3).

^*a*^ The listed components represent representative examples rather than an exhaustive inventory. The table is summarized from previously reported studies (1–3).

As stress persists, the aggresome composition undergoes a significant maturation, evolving from a focused stress-response body into a comprehensive cellular repository (10). This is characterized by the recruitment of a broader range of client proteins, including essential DNA repair enzymes like RecA and central stress-response chaperones such as DnaK. The incorporation of these components signifies a strategic cellular decision to safeguard critical, reusable machinery by inactivating it, thereby committing more deeply to the dormant state. Concurrently, the condensate undergoes a profound physical process of compaction, driven by continued ATP depletion and a resulting increase in the protein-solvent interaction parameter (χ). This is not merely a shrinkage in volume; it is an active densification in which the internal environment becomes significantly more concentrated and crowded. This compaction has critical biophysical consequences. According to Flory-Huggins theory, as the aggresome densifies, the binodal concentration within the droplet shifts, leading to a higher concentration of constituent proteins and RNAs. This creates a self-reinforcing cycle in which the condensed phase becomes increasingly favorable for the partitioning of additional biomolecules.

This evolving composition and physical state collectively drive a critical material state transition. Initially, aggresomes exhibit classic liquid-like properties, characterized by dynamic molecular exchange, rapid FRAP, and the ability to fuse with one another. This liquidity permits rapid disaggregation and facilitates a swift return to metabolism upon stress relief. However, under prolonged stress, the increasing concentration, multivalency of aggregating proteins (particularly structured ribosomal components), and the escalating density of RNA molecules promote a transition to a more gel-like or more solid-like state (10).

Importantly, this transition is not instantaneous but typically occurs progressively as stress persists, reflecting the combined effects of stress duration and intracellular biochemical changes. This liquid-to-gel or solid-like transition is functionally paramount. The gel-like matrix drastically reduces molecular mobility within the aggresomes, potentially transforming it from a dynamic reactor into a static archive. This material hardening imposes a significantly higher kinetic barrier to disaggregation, as the DnaK-ClpB machinery must now work against a rigid, cross-linked structure rather than a fluid one. Consequently, this transition directly deepens the cellular dormancy, increasing the resuscitation lag time and potentially pushing cells into a VBNC state. The aggresome thus functions as a programmable material, whose physical state—orchestrated by stress duration and intensity—calibrates the depth of cellular shutdown. Whether aggresome maturation involves a strict “point-of-no-return” remains unclear; however, it is plausible that fluctuating environments may partially reverse condensate maturation, allowing cells to move back toward shallower dormancy states.

## PHASE PLASTICITY AS A TUNABLE RHEOSTAT FOR BACTERIAL DORMANCY DEPTH

Phase plasticity refers to the ability of condensates to dynamically transition between liquid-like and more solid-like material states. Emerging evidence supports a central hypothesis: the material state of the aggresome functions as a cellular rheostat, directly calibrating dormancy depth. This model reframes bacterial persistence as a physical continuum—spanning from reversible, liquid-like condensates characteristic of shallow dormancy to gel-like assemblies that deepen and prolong metabolic arrest. Liquid aggresomes maintain a shallow dormant state where their intrinsic fluidity enables rapid component exchange and swift dissolution upon stress removal. The maturation of microbial aggresomes—from liquid-like to gel-like states—is influenced by both time and environmental stress, although direct evidence specifically within microbial systems remains scarce. This process is efficiently facilitated by ATP-dependent disaggregases such as DnaK-ClpB, which process proteins within these dynamic condensates to enable rapid resumption of growth (8). In contrast, under sustained stress conditions—such as prolonged starvation, arsenite exposure, or excessive molecular crowding—these aggresomes undergo a phase transition to a gel-like state. Research on analogous systems, such as GRP proteins in tick saliva, while not directly focused on microbial aggresomes, demonstrates clear time-dependent liquid-to-gel transitions mediated by specific molecular interactions (45), suggesting that temporal progression alone can drive maturation even in the absence of continuous stress. This transition reinforces multivalent interactions, effectively trapping essential cellular components, including ribosomal proteins, metabolic enzymes, and mRNAs. The resulting gel-like matrix significantly elevates the energy and enzymatic cost of resuscitation, as disaggregation now requires overcoming substantial structural inertia. Cells precisely tune their position along this liquidity-solidity spectrum through multiple regulatory inputs: by modulating ribosome sequestration to balance translational capacity against stress maintenance requirements; by recruiting specific architectural RNAs that influence condensate properties; and through dynamics of ATP depletion and recovery, which directly govern LLPS stability (8–10).

The evolutionary conservation of this LLPS-driven aggresome pathway across Gram-negative pathogens underscores its role as a fundamental adaptive strategy. It provides a physical basis for managing critical fitness trade-offs—balancing immediate survival under antibiotic or oxidative stress against the future metabolic cost of resuscitation—thereby enabling pathogens to optimize persistence within dynamically hostile host environments.

## THERAPEUTIC TARGETING OF AGGRESOME PLASTICITY IN TREATING PERSISTER CELLS

The growing understanding of aggresome plasticity—governed by LLPS and stress-induced compaction—opens novel therapeutic avenues for combating persistent infections. This paradigm shifts the goal from simply killing bacteria to actively manipulating their physiological state. Therapeutic strategies can be designed to modulate dormancy depth bidirectionally: either by forcing resuscitation to sensitize cells to conventional antibiotics, or by deepening dormancy into an irreversible state that leads to cell death or permanent stasis.

For acute infections (such as urinary tract infections), the “forcing resuscitation” approach aims to dismantle the protective barrier of shallow dormancy by luring persister cells into a metabolically active state, thereby restoring their susceptibility to conventional antibiotics. This can be achieved through several targeted strategies: first, by developing small-molecule agonists that hyper-activate key disaggregase machinery, such as the ClpB-DnaK complex, to artificially boost the “escaping speed,” thereby promoting the dissolution of liquid-like aggresomes and synchronizing persister resuscitation for timed antibiotic treatment; second, by applying compounds that destabilize the condensed phase of aggresomes—such as those interfering with multivalent protein-RNA interactions or ionophores that perturb intracellular ion homeostasis—to induce a phase transition back to a dynamic, liquid-like state; and third, by modulating cellular energy dynamics, either through inducing futile metabolic cycles to increase energy demand or by supplying transient non-replicative energy sources to elevate ATP levels past a critical threshold, thus priming dormant cells for resuscitation.

Conversely, for chronic infections (such as tuberculosis), a “point of no return” strategy aims to therapeutically push persister cells from a reversible, shallow dormancy into a deep, non-viable state (8). This could be achieved through the development of therapeutic agents designed to exacerbate the aggresome’s phase transition, for instance, by promoting excessive protein cross-linking within the condensate or by inhibiting the cell’s minimal maintenance processes, thereby transforming it from a reversible protective body into a permanent, toxic aggregate. A related strategy involves the use of designed peptides that promote the formation of toxic protein aggregates in bacteria, which has been shown to be effective against drug-resistant clinical isolates (46). This strategy could be further potentiated via synergistic lethality with host defenses, where a pro-gelation drug is administered alongside agents that enhance macrophage oxidative bursts; the resulting gel-like aggresomes would be incapable of protecting their sequestered cellular machinery, leading to synergistic oxidative damage and ultimate cell death.

Therapeutic efficacy can be further enhanced through host-directed adjunctive therapy that modulates the host environment to discourage protective aggresome formation (47). A complementary approach involves tempering immunopathology in chronic infections such as cystic fibrosis or tuberculosis, where excessive host inflammatory responses—including rampant macrophage oxidative bursts and neutrophil extracellular trap formation—serve as potent inducers of bacterial dormancy (11); employing host-directed anti-inflammatories to mitigate these responses can reduce the stress signals that trigger aggresome assembly, thereby preventing transitions into deep dormancy and maintaining pathogen susceptibility to antibiotics. Furthermore, for intracellular pathogens like *Mycobacterium tuberculosis* that manipulate phagosomal maturation to survive, pharmacological restoration of normal phagosomal acidification and trafficking can create an environment that favors lethal stress over protective dormancy, effectively undermining the conditions necessary for bacterial persistence.

Beyond direct antimicrobial strategies, the role of aggresomes opens promising avenues for diagnostic and prognostic innovation, positioning them as valuable clinical biomarkers. The abundance, composition, and material state of aggresomes—particularly a high burden of gel-like forms in clinical samples such as sputum or tissue biopsies—could indicate a deeply dormant, treatment-recalcitrant infection, thereby stratifying patients according to relapse risk and signaling the need for tailored anti-persister regimens. Furthermore, integrating advanced imaging techniques capable of detecting aggresome formation *in vivo*, such as super-resolution microscopy or label-free quantitative phase imaging, into clinical practice would enable real-time visualization of the “dormancy burden” of an infection, guiding the transition from conventional antibiotics to persistence-targeting therapies and objectively monitoring treatment response.

However, therapeutic strategies targeting aggresomes must also be tempered by significant biological constraints. First, achieving sufficient specificity to disrupt bacterial aggresomes without perturbing essential host protein quality control systems remains a major hurdle; off-target effects could trigger unintended stress responses or cytotoxicity. Second, bacteria may activate compensatory pathways—such as alternative chaperone networks or redundant toxin-antitoxin modules—that bypass aggresome-dependent survival mechanisms, limiting intervention efficacy (2, 48). Regarding the logic of intervention, fluidizing aggresomes might promote resuscitation and re-sensitization to antibiotics in chronic infections where rapid clearance is desired, whereas hardening them could lock bacteria in a dormant state, potentially reducing relapse in contexts where immune-mediated clearance is robust. Yet, the optimal approach likely depends on infection stage, pathogen species, and host immunity (49, 50). Although therapeutic manipulation of aggresomes is unlikely to represent a near-term clinical intervention, it provides an important conceptual framework for developing strategies to overcome antibiotic tolerance. At present, these ideas should be viewed primarily as conceptual frameworks for guiding future research rather than immediate therapeutic strategies.

Targeting aggresome plasticity represents a frontier in anti-infective therapy, moving beyond the traditional “find and kill” paradigm to a more nuanced “control and eliminate” approach. While significant challenges remain, the ability to strategically manipulate bacterial physiology at the level of biomolecular condensates holds immense promise for finally overcoming the formidable challenge of persistent infections.

## CONCLUSIONS AND EMERGING FRONTIERS

The study of bacterial aggresomes represents a conceptual shift: these structures are not passive deposits of misfolded proteins but adaptive organelles that regulate cellular dormancy through the biophysics of LLPS. In contrast to earlier models that treated protein aggregation largely as a static consequence of ATP depletion, the framework proposed here conceptualizes aggresomes as phase-plastic condensates that form a tunable continuum of dormancy depth. By acting as dynamic reservoirs of essential cellular machinery, aggresomes enable bacteria to modulate metabolic shutdown and recovery trajectories in response to environmental stress.

Importantly, integrating RNA dynamics, material-state plasticity, and stress-dependent maturation significantly extends prior aggregation paradigms. While classical models focused primarily on misfolded protein accumulation, emerging evidence shows that bacterial aggresomes selectively enrich specific mRNAs—particularly longer transcripts—while excluding ribonucleases through electrostatic interactions (10). This selective RNA storage may facilitate rapid translational reactivation following stress relief. Together, these findings suggest that aggresomes function not merely as byproducts of proteostasis failure but as regulated biomolecular condensates that coordinate proteostasis, RNA preservation, and metabolic sensing within a single adaptive system.

Several frontiers remain ripe for exploration. What specific sequence or structural features drive the selective sequestration of proteins into aggresomes? What is the precise role of RNA, beyond mRNA protection, in nucleating and stabilizing condensate architecture? Answering these questions will require the development of new tools, such as *in vivo* LLPS biosensors and single-cell translatomics, to dissect the dynamics of these processes in real time. Unlocking the secrets of aggresome biology promises not only fundamental insights into bacterial resilience but also new strategies to overcome one of the most challenging problems in modern medicine: antibiotic tolerance.
